# The Army and the Medical Profession

**Published:** 1915-03-27

**Authors:** 


### The Hospital

The Workers' Newspaper of Administrative Medicine and Institutional Life, Administration, National Insurance and Health.

No. 1501, Vol. LVII. SATURDAY, MARCH 27, 1915. PRICE ONE PENNY.

### THE ARMY AND THE MEDICAL PROFESSION.

Whatever else may have been proclaimed and emphasised by the war it has been made very evident that the Army cannot get on without doctors. Times have indeed changed since the military surgeons—not, after all, so very long ago—were regarded as little better than well-dressed camp followers, and their claims to official rank were ignored or even treated with contempt. All this is a bad past, and we have done with it. Nowadays the co-operation of all branches of the Army is recognised as essential to victory, and the doctors will acknowledge that their services have been generously recognised alike by the soldiers and the country. The difficulty has now come to be that there are not enough of them. Instead of being superfluous and of secondary importance they are pushed into prominence, and larger and larger numbers are required.

The Director-General's request addressed to the profession, and calling for a greater amount of help, is a document of very great importance. It has the responsibility of an authoritative and official statement, and it is hardly less weighty than Lord Kitchener's announcement in reference to the shortage of war munitions. There is no dubiety or ambiguity about such a phrase as "the need for medical men, both for home and foreign service, is acute," and, especially in view of the reserve which marks official utterances, there is no doubting the meaning of the sentence "every man who can arrange for his work to be done at home should come forward as early as possible if we are to keep up an adequate supply of medical attendance to our armies in the field." All this is wholesome plain-speaking, and is very much to be commended. When we are told what is wanted of us we know what we have to give. Somehow or other, then, an increased volume of medical service has to be supplied for the work of the Army, and the only question is how this can be best effected.

Now it is certain that the reserves of the medical profession have already been considerably invaded, and it may be doubted whether any further number of doctors can be detached for Army purposes without prejudice to the necessities of the civil population. As is well known, hospitals have great difficulty in procuring resident staffs, and in many cases private practitioners are overdriven by the added responsibility of work left behind by colleagues summoned to military duties. We agree that in the circumstances the soldiers have the first claim, and that whoever else suffers neglect it must not be they. But neglect in either direction would be a truly lamentable state of affairs, and every effort must be made to avoid it. The enormous probability is that it will be avoided if the resources of the profession are carefully and efficiently organised, and what is very much to be desired at the moment is the appearance of some influence by which this organisation can be effected. The goodwill of individuals is beyond question, but there is a sad need of leadership and arrangement. The position is surely one in which some of the professional organisations ought at once to set their machinery in motion, for so far, at least in their corporate capacity, they seem to have done nothing. Some of them are very fond of emphasising their "royal" character, and whatever else this may mean it certainly ought to imply a special readiness for public and patriotic duty. Men are willing and anxious to help to the full measure of their capacity, but they need counsel and direction and, above all, organisation, and these ought to be forthcoming from the profession itself. If colleges and associations and the rest have no help or leading to give in such a crisis as the present, it may be difficult to find a justification for their existence when the nation, at the close of the war, proceeds to overhaul its institutions.

The real difficulty of the situation appears to be some measure of conflict between the wishes of the War Office and the wishes of many civil practitioners. Naturally the War Office desires to deal with men who take commissions in the R.A.M.C., and who are therefore "soldiers" in the technical sense of this word. On the other hand, many practitioners have a not unnatural fear that if they once become incorporated in the official machine they will never know how far it may carry them. For domestic and other reasons it is impossible for them to give up their means of livelihood on the strength of an appointment which has no permanent quality; and this consideration has to be weighed as well, also, as the claims of the civil population. Take, again, the responsibilities of many who hold hospital appointments, and who could not push these to one side without a plain neglect of duty. What is wanted is some plan which will leave all these activities to be continued, and will at the same time secure proper medical attention for the soldiers. There are only two possibilities. The one is that the War Office should be willing to accept part-time duty from practitioners who retain their civil status; the other, that the conditions of service should be so clearly defined that a practitioner in accepting a commission would feel certain that there was no risk of more being demanded from him than he had undertaken to give. There is no doubt that many members of the profession would, if the demand was made on them, find, say, two hours a day for military duty in some form or other, provided that they are left free otherwise to continue their civilian work. Granted this condition, it is a matter of complete indifference to them whether they are or are not made "soldiers." It is, of course, easy to understand the difficulties of the authorities in reference to discipline and to precedence when dealing with a scheme which includes both military and civil practitioners. If the only way of surmounting these difficulties is that still more members of the profession must join the R.A.M.C., the position must be accepted. But let this arrangement be made in such a fashion as to conserve the interests of the individual and the welfare of civil practice.

